# The Extracellular Matrix-Derived Biomarkers for Diagnosis, Prognosis, and Personalized Therapy of Malignant Tumors

**DOI:** 10.3389/fonc.2020.575569

**Published:** 2020-12-18

**Authors:** Elena V. Petersen, Daria A. Chudakova, Ekaterina Yu. Skorova, Vladimir Anikin, Igor V. Reshetov, Ospan A. Mynbaev

**Affiliations:** ^1^ Department of Molecular and Bio Physics, Moscow Institute of Physics and Technology, Dolgoprudny, Russia; ^2^ School of Biological Sciences, University of Auckland, Auckland, New Zealand; ^3^ Harefield Hospital, The Royal Brompton and Harefield Hospitals NHS Foundation Trust, Harefield, United Kingdom; ^4^ Department of Oncology and Reconstructive Surgery, Sechenov Medical University, Moscow, Russia

**Keywords:** biomarkers, cancer, tumor microenvironment, extracellular matrix, extracellular vesicles, 3D cell culture, personalized therapy

## Abstract

The tumor biomarkers already have proven clinical value and have become an integral part in cancer management and modern translational oncology. The tumor tissue microenvironment (TME), which includes extracellular matrix (ECM), signaling molecules, immune and stromal cells, and adjacent non-tumorous tissue, contributes to cancer pathogenesis. Thus, TME-derived biomarkers have many clinical applications. This review is predominately based on the most recent publications (manuscripts published in a last 5 years, or seminal publications published earlier) and fills a gap in the current literature on the cancer biomarkers derived from the TME, with particular attention given to the ECM and products of its processing and degradation, ECM-associated extracellular vesicles (EVs), biomechanical characteristics of ECM, and ECM-derived biomarkers predicting response to the immunotherapy. We discuss the clinical utility of the TME-incorporating three-dimensional *in vitro* and *ex vivo* cell culture models for personalized therapy. We conclude that ECM is a critical driver of malignancies and ECM-derived biomarkers should be included in diagnostics and prognostics panels of markers in the clinic.

## Introduction

Cancer remains one of the leading causes of deaths globally with a strong tendency to become the “number one killer disease” in the 21st century (1). Despite the recent achievements in understanding how malignant tumor arise and develop there are many unique aspects of tumorigenesis which are not fully understood, one of them is how the tumor microenvironment (TME) orchestrates a wide array of events in the tumor initiation and progression. The studies focusing on the role of TME in cancer initiation and progression may identify novel therapeutic targets and biomarkers derived from within the TME, with clinical translational potential.

A biomarker is a biological characteristic that can be identified and objectively evaluated as an indicator of a normal or pathological biological process (2) and may serve for various clinical purposes (3, 4). The prognostic biomarkers can predict favorable or unfavorable outcome of disease irrespective of the therapy, predictive biomarkers may foresee favorable or unfavorable response to the therapy. It is apparent now that only genomic biomarkers are not clinically informative enough, and the set of available diagnostic tools should be expanded (5). The growing number of studies demonstrates that biomarkers identified not only in the tumor cell itself, but also within the TME are valuable diagnostic tools (6–8) (**Supplementary Table 1**). These biomarkers include bio-mechanical characteristics of ECM, structural components of ECM, products of ECM biosynthesis, processing, degradation, proteinase inhibitors, as well as activators, circulating EVs, cytokines, and others. There are a number of techniques allowing detection of such biomarkers in a clinical setting, such as ELISA (9), microscopy and imaging analysis (10), mass-spectrometry (11) including MALDI imaging mass-spectrometry applied to analysis of formalin-fixed paraffin-embedded tissues (12), immunohistochemistry (13), Western blotting (14), RT-qPCR (15), and others.

In the present review, we will provide a framework for understanding the role of TME-associated biomarkers in cancer pathogenesis and discuss their clinical utility in precision oncology, with special emphasis on biomarkers predicting response to immunotherapy. Particular focus is given to the ECM-derived protein markers, EVs and their molecular cargo, biomechanical characteristics of ECM, and ECM-incorporating 3D cell culture models for translational oncology.

## ECM Components as Cancer Biomarkers

ECM is an extracellular three-dimensional (3D) maze-like structure formed by a variety of macromolecules such as proteins, proteoglycans, glycoproteins, polysaccharides, and others (16, 17). It also contains multitude of matrix-stored regulatory and signaling biomolecules, such as growth factors and cytokines, circular RNAs (circRNAs), and miRNAs within the TME-associated exosomes, and others (18, 19). Structurally, the ECM comprises the basal membrane and the interstitial tissue. The components of ECM, also referred to as “matrisome” (20), are produced by the cells of several types, predominantly fibroblasts (21). Interactions of cell surface receptors with the components of ECM enable cell-ECM adhesion, which is vital for many types of anchorage-dependent cells (22). ECM has a plethora of functions—it creates a niche for stem cells and regulates intercellular chemical and mechanical signaling networks, angiogenesis, innate and adaptive immune response, and migration and invasion of the cells (23–25). All this makes the ECM one of the key regulators of cancer progression and response to the therapy, capable of modulating fundamental hallmarks of cancer (26).

The molecular composition, mechanical properties of ECM, its infiltration by immune cells and stromal cells is heterogeneous and immensely diverse in different types of tumor tissues. To accommodate the specific needs of the tumor, both cancer cells and tumor-associated stromal cells modify ECM by producing and secreting ECM-modifying enzymes. For example, fibroblasts associated with tumor tissue (cancer-associated fibroblasts, CAFs) and tumor-associated macrophages (TAMs) modify ECM to create a metastasis-permissive environment (27, 28). Many components of ECM are deregulated in cancer, and some oncogenic macromolecules within the tumor ECM are upregulated whereas tumor-suppressors are downregulated (29) (**Supplementary Table 1**). The analysis of expression of 820 matrisome genes across a panel of 32 malignant tumors has identified universal pan-cancer gene signatures which supposedly might be used for diagnostics (30). Recent study of the changes in the matrisome during the cancer progression identified expression patterns of the 22 genes associated with shorter overall survival of patients with ovarian and several other solid tumors (31). Several independent attempts have also been made to characterize the profile of ECM-derived biomarkers for a particular type of cancer and identify cancer-specific markers for clinical application (**Supplementary Table 1**).

Some ECM-derived peptides, termed “matrikines” or “matricryptines”, have cytokine-like activity (32). The matricryptines are generated by the structural or enzymatic modification of ECM resulting in exposure of the biologically active and previously hidden (“cryptic”) sites. It has been suggested recently that cryptic collagen elements serve as signaling hubs regulating tumor metastasis and growth (33). ECM may also evolve releasing biologically active substances, including matrikines, which may be used as “protein fingerprint” of cancer. One of them is Tumstatin derived from collagen type IVα3 and described as a biomarker for non-small-cell lung cancer (NSCLC) (34).

Importantly for transnational oncology, ECM-derived biomarkers may reflect response to therapy, including immunotherapy. Whereas the role of stromal cells within TME in immune response is comprehensively studied and reviewed elsewhere (35), the role of ECM and products of its modification as biomarkers of tumor response to immunotherapy is not well known. Recent study demonstrated that tumor matrisome gene signatures are predictive biomarkers of resistance to ICT immunotherapy (36). Versican-derived matrikine versikine is a biomarker of tumor response to immunotherapy (37) and regulator of tumor infiltration by T-cells (38, 39) [notably, versican itself is upregulated in cervical cancer and leiomyosarcoma (40, 41)]. In patients with stage IV melanoma, collagen-derived biomolecules RO-C3, C1M, C3M, and C4M are biomarkers of poor response to the therapy with immune checkpoint inhibitor (ICI) ipilimumab (42). In patients with metastatic melanoma, blood-based biomarkers of type III collagen turnover are associated with worse overall survival and progress-free survival following PD-1 inhibition immunotherapy (43). In a clinical scenario, the ECM-turnover associated with the response of melanoma to immuno-therapy might be assessed in a “liquid biopsy” (44), and allows to stratify patients with metastatic melanoma according to their response to ICI therapy (45). Finally, many protein biomarkers of tumor invasiveness localized in ECM have been identified (comprehensively reviewed in (46).

Aforementioned, many soluble ECM-derived molecules arising from within a solid tumor can be found in a peripheral blood, are detectable using routine laboratory methods such as immunoassays (42, 43), and may therefore be used as a non-invasive “liquid biopsy” biomarkers. This makes them very attractive for use in clinics (47), the only limitation of their use being sensitivity and specificity of the immunoassay.

## Mechanical and Physical Properties of ECM as Cancer Biomarkers

Mechanotransduction, also known as mechanosignaling, is a process through which cells initiate a biochemical process in response to mechanical signals. The stiffness, topology, and other mechanistic characteristics of the ECM are critical drivers and regulators of the tumor progression, affecting cancer cell biology *via* the mechanotransduction [comprehensively reviewed in (48–50)] and therefore can be used as biomarkers of malignancy (51, 52). The phenomenon of durotaxis (directed migration of the cells in response to the gradient of stiffness of the substrate) also plays an important role in tumorigenesis (53, 54).

The biomechanical properties of the ECM dynamically change over the course of the disease and differ between tumor and matched normal tissue. In many types of solid tumors, ECM within the tumor tissue is more rigid than ECM of matched non-tumorous tissue (55) mostly because of the elevated deposition and cross-linking of collagen type I, which can be detected by the imaging or manual examination. Such stiffness of the ECM induces epithelial-to-mesenchymal transition (EMT) of the cancer cells, thus resulting in a metastatic phenotype, for example, in pancreatic ductal adenocarcinoma (56) and in hepatocellular carcinoma (57). On the other hand, ovarian cancer cells undergo EMT on softer substrates (58), and soft matrices enhance cancer stem cell phenotype in hepatocellular carcinoma (59). This should be considered then developing therapeutic approaches aimed to modify softness/rigidity of the ECM and targeting its mechanical features (60, 61).

The overall role of the biomechanical properties of ECM in several types of cancer, for example, esophageal cancer (62), ovarian cancer (63), and colorectal cancer (64), has been comprehensively reviewed recently (**Supplementary Table 1**). The stiffness of the ECM can also be a biomarker predicting response to the chemotherapy; for example, it has been shown that in case of pancreatic ductal adenocarcinoma it induces chemoresistance to paclitaxel, but not to gemcitabine (56). Furthermore, the mechanical characteristics of ECM play a role in immune oncology and therefore might be a biomarker of response to immunotherapy. For example, stiffness of ECM modulates PD-L1 expression in lung cancer (65) and breast cancer cells (66) and regulates activity of T-cells within the tumor tissue (67).

There are several powerful tools and approaches available to assess mechanical and physical properties of TME, for example, high resolution Atomic Force Microscopy (AFM), Scanning Electron Microscopy (SEM), Spatial Light Interference Microscopy (SLIM), and others (64, 68–70). The Multiphoton Microscopy and Second Harmonic Generation (SHG) imaging can be applied to analyze “evolution” of collagen within the ECM during tumor progression (71). That said, non-invasive imaging techniques, such as Ultrasound Elastography, Magnetic resonance elastography, Magnetic Resonance Imaging (MRI), and others might still be a good option for assessing biomechanical characteristics of the tumor tissue in clinic (72–74).

## Extracellular Vesicles as Cancer Biomarkers and Regulators of TME

Within the TME, cells communicate *via* different mechanisms including extracellular vesicles (EVs). EVs are carriers of a biologically active molecular cargo (lipids, nucleic acids, proteins, mRNA, miRNA, circRNA, lncRNA, and others). As some of the contents of EVs may modulate ECM [for example, matrix-remodeling enzymes (75)] or participate in a cross-talk of the cancer cell with stromal cells, thus contributing to chemotherapy resistance or metastasis, there is a possibility to use EVs within a TME as a therapeutic targets and therapeutic biomarkers. Moreover, the possibility to detect tumor-derived EVs in a bloodstream makes them attractive for use in a clinical setting (76).

In the context of ECM, there is a subset of matrix-bound nanovesicles (MBVs) (77, 78) present within the ECM rather than in biological fluids. They are embedded into the ECM, express surface antigens that are commonly found on exosomes, and can be isolated from the matrix only by methods of enzymatic digestion of ECM scaffolds (77). Their molecular cargo comprises miRNAs and is capable of changing the phenotype of the cells exposed to the contents of MBVs, for example, affecting the phenotype of macrophages (78). MBVs are integral and distinct components of ECM, and their content is unique to cellular origin (78). Recently, it has been demonstrated that MBVs can suppress pro-inflammatory signaling in microglia and astrocytes (79). So far, the literature exists only on MBVs found in non-timorous ECM, but we propose that tumor-specific MBVs may also be found. If molecular cargo of MBVs is cell type-dependent and unique to cellular origin, as demonstrated by Hussey et al., the MBVs derived from tumor ECM most likely will also have unique and tumor-specific characteristics. Further studies on this subject should be carried out on various types of malignancies to assess feasibility of using MBVs as potential biomarkers.

Finally, the promising avenue in translational oncology is a possibility to study EVs *in vitro* using cell culture models to identify and characterize novel cancer biomarkers. It has been demonstrated recently that there are cell culture-dependent differences in the content and production of EVs (80). The essential molecular cargo components of EVs secreted by cancer cells cultured *in vitro* in two-dimensional (2D) or 3D format are different, and EVs from 3D culture have much higher similarity to the EVs secreted *in vivo* by tumor tissue (81), and the spectrum of small RNAs in EVs derived from cells in 3D culture has approximately 96% similarity to EVs from cancer patient’s plasma (81). This provides a rationale for developing 3D cell culture-based *in vitro* model systems for cancer biomarkers identification.

## 3D Cell Culture Models Incorporating TME as a Testing System in Translational Oncology and Personalized Therapy

Over the past decades, significant progress has been made in developing *ex vivo* models that recapitulate *in vivo* tumor characteristics including response to the therapy. It is apparent now that *in vitro* 2D culture of cells on glass or plastic is not an accurate model of *in vivo* “biological reality”. Moreover, mono-culture of cancer cells is a less accurate model compared to the co-culture of cancer cells and stromal cells. Adding to this complexity, compared to the 2D culture, the *in vitro* 3D cell culture models, especially the models including ECM, more closely resemble *in vivo* TME, better reproduce a variety of conditions such as inter-tumor heterogeneity of hypoxia *in vivo*, and more closely resemble a patient’s response to the therapy compared to a 2D mono-culture, as have been demonstrated in many studies.

Currently, 3D systems with tunable ECM stiffness, bio-printed 3D cell culture systems incorporating TME, systems based on 3D culture of patient’s tumor tissue, and systems utilizing decellularized ECM from the patient’s tumor have been established (82–85). Such systems have a clear potential for use in translational oncology. For example, a 3D *in vitro* model of pancreatic ductal adenocarcinoma (PDAC) mimicking mechanical properties of the TME potentially allows more accurately distinguish between pancreatic cancer and pancreatitis (86). A host of technologies and tools have been developed to study the impact of ECM biomechanics on a cell behavior in a variety of 3D cell culture models [comprehensively reviewed in (87)].

Three-dimensional cell culture systems also have proven to be a “biomarkers goldmine”—a valuable tool for biomarker identification (88). For example, using 3D culture model with decellularized ECM scaffolds (dECM) allowed to identify full-length Collagen VI as a driver of breast cancer cell invasion in obesity and metastasis (89). In colorectal cancer, the patterns of expression of miRNA dependent on 3D microenvironment have been characterized, and one of them (miR-142-5p) was identified as a theranostic biomarker (90). As applied to clinical scenarios, the 3D cell culture models incorporating TME are referred to as a “patient’s avatar” (91) and “patient-on-a-chip” (92) models, and can allow to identify biomarkers of individual response to the therapy. For example, patient-derived 3D organoid culture of breast tumor was utilized to choose personalized chemotherapy (93). The feasibility of the automated real-time pharmacokinetic profiling in 3D tumor models has been demonstrated (94), and 3D micro-tumor platform comprising ECM-derived hydrogel and patient-derived colorectal tumor tissue has been created for high-throughput screening of the chemotherapies in a patient-specific format (95). Further development of ECM‐mimicking scaffolds and 3D bio-printing (comprehensively reviewed in (96)) can potentially assist in personalized therapy, although it has been suggested that some 3D cell culture models are rather too complex for routine implementation in clinics at this stage (97), and clinical use of such models would require a high level of methodological (as well as clinical) validation (98). In the next few years, we expect to see a growing number of publications in this emerging field of research.

**Figure 1:**
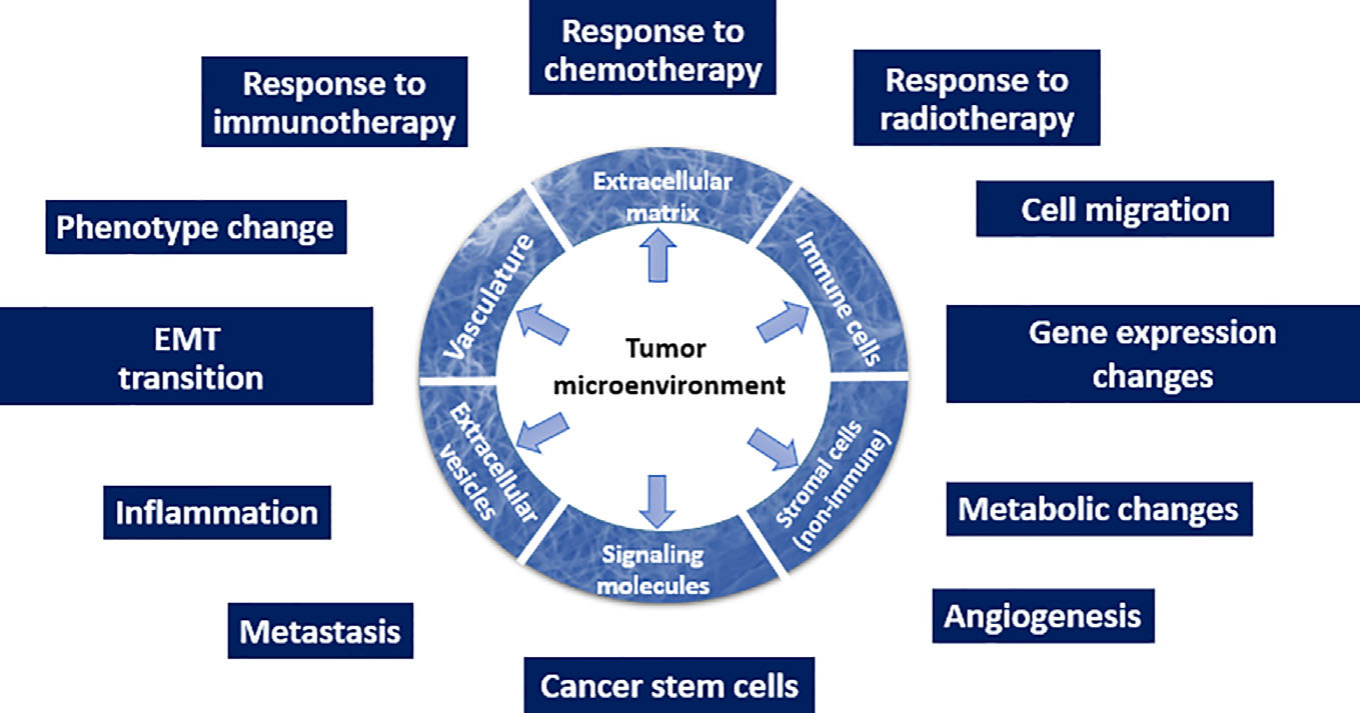
Schematic depiction of the TME and its multiple roles in the tumor initiation and progression.

Overall, the recognition that TME is one of the drivers of malignancy (**Figure 1**) changes the current approach to how malignant tumors will be diagnosed and treated. Here, we emphasize that all types of the TME components depicted in **Figure 1** (such as ECM and its mechanical or biological characteristics, EVs, phenotype of stromal cells, and others) have a potential to serve as biomarkers.

## Conclusion

ECM-derived biomarkers have a great potential in translational oncology and in clinical use. Hitherto, many novel biomarkers arising from within the ECM have been identified, although the clinical utility of many of them remains to be assessed. Based on a multitude of recent studies, we conclude that TME should be included into the *in vitro* and *ex vivo* models for cancer drug development and personalized therapy. In particular, 3D cell culture models incorporating TME and tumor-specific mechanistic characteristics of ECM, such as stiffness and topology, are more accurate and physiologically relevant models of the tumor compared to the traditional cell culture or animal xenograft models.

## Author Contributions

All authors contributed to the article and approved the submitted version. Conception of the study and critical revision of the manuscript: DC, EP, IR, OM, and VA. Literature collection, analysis, and **Supplementary Table** preparation: ES. Literature collection, analysis, and manuscript writing: DC and EP.

## Funding

This work was partially supported by the Russian Science Foundation under grant No.18-15-00391. 

## Conflict of Interest

The authors declare that this research was conducted in the absence of any commercial or financial relationships that could be construed as a potential conflict of interest.
