# Supplementary material for: The Extracellular Matrix-Derived Biomarkers for Diagnosis, Prognosis, and Personalized Therapy of Malignant Tumors
Source: Front Oncol. 2020 Dec 18;10:575569. doi: 10.3389/fonc.2020.575569 (PMC7793707; doi:10.3389/fonc.2020.575569)
Supplement: Supplementary file 1 [file DataSheet_1.doc]

**Supplementary Table 1. Selected TME-associated biomarkers.** PM – prognostic and/or predictive marker, DM – diagnostic marker, F – favorable prognosis, unF - unfavorable prognosis, MMPs – matrix metaloproteinases. THPA - expression in cancer, data from The Human Protein Atlas (www.proteinsaltlas.org).

| **Name of Biomarker** | **Type** | **Biomarker's function in ECM** | **Type of cancer** |
| --- | --- | --- | --- |
|
|
| AFP | PM | ECM binding | Hepatocellular carcinoma; PM (99), not PM (THPA) |
| VNN1 | PM | ECM binding | Renal cancer (unF) (THPA) |
| LOX | PM | ECM binding, modification | Bone marrow cancer (100), renal cancer (unF), urothelial cancer (unF), liver cancer (unF) (THPA) |
| Galectin-1 | PM / DM | ECM binding, modification | Thyroid carcinoma – DM (101), renal cancer (unF), endometrial cancer (F), liver cancer (unF), urothelial cancer (unF) (THPA) |
| Galectin-3 | PM / DM | ECM binding, modification | Thyroid carcinoma, prostate cancer, colorectal cancer (102) - PM, (THPA) - not PM |
| ADAM8 | PM | ECM binding, remodeling via MMPs | Breast cancer (103),(104),(105) |
| BMP | PM | ECM binding, remodeling via MMPs | Clear Cell Renal carcinoma (progression) (106) |
| FBLN2 | PM | ECM binding, structural | Liver cancer (F), urothelial cancer (unF), endometrial cancer (unF) (THPA) |
| MMRN1 | PM | ECM binding, structural | Renal cancer (unF), stomach cancer (unF) (THPA) |
| CTSB and CTSL | PM | Enzyme, ECM degradation | Squamous Cell Carcinoma of the Head and Neck (107) |
| CTSK | PM | Enzyme, ECM degradation | Prostate cancer (108) |
| C3M | PM | Product of ECM degradation | Breast cancer (109) |
| C4M | PM | Product of ECM degradation | Breast cancer (109) |
| COL10A1 | DM | Product of ECM degradation | Breast carcinoma(110), gastric cancer (111) |
| COL11A1 | DM | Product of ECM degradation | Breast carcinoma (110), non-small cell lung cancer (112) |
| COMP | DM | Product of ECM degradation | Breast carcinoma (110) |
| CXCL12 | PM | chemokine | Neurofibroma PM (113), several types of solid tumours (114), colon cancer (115) |
| ELM12 | DM | Product of ECM degradation | Non-small cell lung cancer (116) |
| ELM7 | DM | Product of ECM degradation | Non-small cell lung cancer (116) |
| EL-NE | DM | Product of ECM degradation | Non-small cell lung cancer (116) |
| EL-CG | DM | Product of ECM degradation | Non-small cell lung cancer (116) |
| STAG3 | PM | ECM degradation | Cervical cancer (F) (THPA) |
| BMP1 | PM | ECM remodeling | renal cancer (unF) (THPA) |
| FAP | PM | ECM modification | Head and Neck cancer (unF) (THPA) |
| KIT | PM | ECM modification | Renal cancer (F) (THPA) |
| CDH1 | PM | ECM organization | Paget's disease, Bowen's disease (in situ Squamous Cell Carcinomas) (117) |
| CTSD | PM | ECM remodeling | Glioma (unF) (118) |
| YAP1 | PM | ECM remodeling | Pancreatic cancer (119) |
| ARRB2 | PM / DM | ECM remodeling | Lung cancer (120), ovarian cancer , prostate cancer (unF) and cervical cancer (F)(THPA) |
| BGN | PM | ECM remodeling | Renal cancer (unF) (THPA) |
| BST2 | PM | ECM remodeling | Breast cancer (F), testis cancer (unF), renal cancer (unF) (THPA) |
| FAT1 | PM / DM | ECM remodeling | Colorectal cancer (121), lung cancer (unF) (THPA) |
| MMP1 | PM | ECM remodeling | Lung cancer metastasis (122). Renal cancer (unF), liver cancer (unF), cervical cancer (unF) (THPA) |
| MMP 2 | PM | ECM remodeling | Cutaneous Basal and Squamous Cell Carcinomas (123),(124); Squamous Cell Carcinoma of the Head and Neck (125) – PM, (THPA) - not PM |
| MMP3 | PM | ECM remodeling | Cervical cancer (unF) pancreatic cancer (unF) (THPA) |
| MMP7 | PM | ECM remodeling | Lung cancer (unF), liver cancer (unF) (THPA) |
| MMP9 | PM | ECM remodeling | Cutaneous Basal and Squamous Cell Carcinomas (123), renal cancer (unF), endometrial cancer (F), liver cancer (unF) (THPA) |
| MMP10 | PM | ECM remodeling | Urothelial cancer (F) (THPA) |
| MMP11 | PM | ECM remodeling | Renal cancer (unF) (THPA) |
| MMP12 | PM | ECM remodeling | Liver cancer (unF) (THPA) |
| MMP14 | PM | ECM remodeling | Ovarian cancer (unF) (THPA) |
| MMP15 | PM | ECM remodeling | Urothelial cancer (F), renal cancer (F) (THPA) |
| MMP19 | PM | ECM remodeling | Renal cancer (unF) (THPA) |
| MMP25 | PM | ECM remodeling | Head and Neck cancer (F) (THPA) |
| MMP28 | PM | ECM remodeling | Pancreatic cancer (unF) (THPA) |
| SPON2 | PM | Secreted ECM protein | Pulmonary adenocarcinoma (126) |
| PDGFRA | PM | ECM remodeling | Head and Neck cancer (F), renal cancer (unF) (28) |
| uPA | PM | ECM remodeling | Breast cancer (127),(128),(129); Squamous Cell Carcinoma of the Head and Neck (130),(131) |
| TIMP3 | PM | ECM remodeling (MMPs inhibitor) | Ovarian cancer (unF), renal cancer (F), urothelial cancer (unF) (THPA) |
| ALDH1 | PM | ECM remodeling via MMPs | Schwannomas and neurofibromas (132) |
| EGFL6 | PM | ECM signaling | Head and Neck cancer (F), ovarian cancer (F) (THPA) |
| miR-497-5p | Potential PM/DM | Exosomal miRNA | Pediatric glioma stem cells (133) |
| miR-675 | PM | Exosomal miRNA | Metastatic osteosarcoma (134) |
| miRNA-191 | DM | Exosomal miRNA | Pancreatic neoplasm (135) |
| VTN topology | PM | Mechanical properties | Neuroblastoma (136) |
| ECM stiffness | PM | Mechanical properties | Breast cancer (137)(unF), neuroblastoma (138). Pancreatic ductal adenocarcinoma PTM of chemoresistance to paclitaxel (56) |
| ECM softness | PM | Mechanical  properties | ovarian cancer (58) (unF), neuroblastoma (138) |
| COL4A1 | PM | structural | Renal cancer (unF), cervical cancer (unF) (THPA) |
| COL6A2 | PM | structural | Renal cancer (unF) (THPA) |
| COMP | PM | structural | Renal cancer (unF), colorectal cancer (unF), endometrial cancer (unF), urothelial cancer (unF) (THPA) |
| DCN | Potential PM/DM | structural, regulatory | Colon cancer (139), cervical squamous cell carcinoma (140) |
| DSG | PM | structural | Urothelial cancer (unF) (THPA) |
| DSC2 | PM | structural | Urothelial cancer (unF) and lung cancer (unF) (THPA) |
| ECM1 | PM | structural | Renal cancer (unF), urothelial cancer (unF), pancreatic cancer (unF) (THPA),  bladder canceer (141) |
| ECM2 | PM | structural | Renal cancer (unF) and liver cancer (F) (THPA) |
| EFEMP1 | PM | structural | Endometrial cancer (unF), urothelial cancer (unF), renal cancer (unF) (THPA) |
| EFEMP2 | PM | structural | Renal cancer (unF) (THPA) |
| ELN | PM | structural | Thyroid cancer (unF) (THPA) |
| EMILIN1 | PM | structural | Renal cancer (unF), pancreatic cancer (unF), renal cancer (unF) (THPA) |
| FN1 | PM | structural | Ovarian cancer (13) |
| ISLR | PM | structural | Renal cancer (unF) (THPA) |
| LAMC1 | PM | structural | Urothelial cancer (unF) (THPA) |
| MFAP5 | PM | structural | Stomach cancer (unF) urothelial cancer (unF) (THPA) |
| mucin-5AC | PM / DM | structural | Pancreatic adenocarcinomas (THPA), (142) |
| PXDN | PM | structural | Renal cancer (F) (THPA), prostate cancer (143) |
| POSTN | PM | structural | Ovarian cancer (13), colon cancer (144) |
| VCAN | PM | structural | Lung cancer (THPA)(145) |
| TNC | PM | structural, regulatory | Lung cancer (146), breast cancer (147), lung cancer metastasis (148), colorectal cancer (149), pediatric brainstem glioma (150) |
